# Icb-1 gene polymorphism rs1467465 is associated with susceptibility to ovarian cancer

**DOI:** 10.1186/1757-2215-7-42

**Published:** 2014-04-23

**Authors:** Susanne Schüler, Claus Lattrich, Maciej Skrzypczak, Tanja Fehm, Olaf Ortmann, Oliver Treeck

**Affiliations:** 1 Department of Gynecology and Obstetrics, University Medical Center Regensburg, Regensburg, Germany; 2 Second Department of Gynecology, Medical University of Lublin, Lublin, Poland; 3 Department of Gynecology and Obstetrics, University of Tübingen, Tübingen, Germany

**Keywords:** Ovarian cancer, *icb-1* gene, Single nucleotide polymorphism, Case control study

## Abstract

In this study, we tested the hypothesis that single nucleotide polymorphisms (SNPs) of differentiation-associated human gene *icb-1* (C1orf38) may be associated with ovarian cancer susceptibility. For this purpose, we compared the genotype and allele frequencies of the SNPs rs1467465 and rs12048235 in a group of 184 ovarian cancer patients with a control group of 184 age- and gender-matched women without any malignancy. Genotype-phenotype association revealed that A allele of SNP rs1467465 was more frequent in ovarian cancer patients than in the control group (0.40 vs. 0.33, OR 1.37, 95% CI 1.013-1.853, p = 0.04). After analysis of allele positivity we observed that A-positive genotypes were more frequent in the ovarian cancer group (0.65 vs. 0.53, OR 1.63, 95% CI 1.072-2.483, p = 0.02). Furthermore, the heterozygous genotype of rs1467465 was found to be more frequent in the patients group (0.50 vs. 0.41, OR 1.63, 95% CI 1.045-2.045, p = 0.03). No significant results were obtained with regard to SNP rs1204823. Our data suggest, that SNP rs1467465 of human gene *icb-1* might affect susceptibility to ovarian cancer.

## Introduction

Ovarian cancer is the most lethal gynecological malignancy and the sixth most common cancer among women in industrialized countries [1]. Because of its potential for aggressive local invasion and the lack of sensitive early screening methods, around 75% of all ovarian cancers are diagnosed at an advanced stage. Despite extensive research during the last decades, the etiology and pathogenesis of this tumor entity is only partly understood. Binding of steroid hormones like estrogens to their receptors like estrogen receptor α (ERα) is known to stimulate growth of ovarian cancer cells [2,3]. Recently we reported interaction between ERα and differentiation-associated gene *icb-1* (Themis2, C1orf38) in ovarian cells [4,5]. *Icb-1* is a vertebrate gene located on human chromosome 1 (1p35.3), which was identified and cloned by our group in an attempt to analyze gene expression changes during in vitro differentiation of endometrial tumor cells [6]. Recent studies suggested *icb-1* to act as a tumor suppressor in ovarian cancer - its knockdown accelerated growth of various ovarian cancer cell lines and led to upregulation of ovarian cancer markers like CLDN16 and KLK10 [7]. Icb-1 seems to suppress progression of ovarian cancer by inhibition of oncogenic pathways activated by ERα. Thus, the individual level of *icb-1*expression, which can be assumed to result from different epigenetical, but also genetical factors like single nucleotide polymorphisms (SNPs), might affect ovarian cancer risk.

Today, only 5–10% of ovarian cancer cases have been shown to be hereditary [8]. However, further polymorphisms in crucial genes are expected to affect susceptibility to ovarian cancer [9]. Single nucleotide polymorphisms (SNPs) are the most frequent sequence variations in the human genome. SNPs located in exon regions may alter protein function, whereas SNPs in the gene promoter can modify gene expression levels [9-15]. In the last years, a multitude of genotype-phenotype association studies have been published examining the significance of randomly chosen SNPs in different hormone-dependent diseases [12,16-20].

To test the relevance of two SNPs of *icb-1* gene for susceptibility to ovarian cancer, we genotyped 184 women with ovarian cancer and just as many women without any malignancy.

## Patients and methods

### Patients

In this study we used blood samples from 184 Caucasian women with sporadic ovarian cancer and a median age at diagnosis of 60.7. The gender-matched control group contained the same number of Caucasian women without any malignancy at the beginning of the study and a median age at inclusion of 60.8. We included ovarian cancer blood samples collected in the Department of Gynecology and Obstetrics of the University of Regensburg, serum samples from the Department of Gynecology and Obstetrics of the University of Tübingen, Germany and further blood samples from the Second Department of Gynecology of the University School of Medicine of Lublin, Poland. Generally, Caucasian women with sporadic ovarian cancer and available information on grading, stage, and histological subtype from 2002 to 2012 were included. The histopathological characteristics of the patients are shown in Table 1. The retrospective study was approved by the institutional review board “Ethikkommission der Universität Regensburg and by the Institutional Review Boards of the Universities Tübingen and Lublin”.

**Table 1:** Staging and histopathological characteristics of ovarian cancer cases (n = 184)

**Characteristics**	**Patient numbers**
**FIGO staging**	
Stage I	38
Stage II	13
Stage III	89
Stage IV	37
unknown	7
**Histological subtype**	
serous	126
endometrioid	36
mucinous	17
clear cell	7
transitional cell	2
undifferentiated	8
**Histological grade**	
G1	24
G2	49
G3	111

### SNP analysis

SNPs in the *icb-1* gene were selected using the internet web sites http://www.genecards.org. and http://www.ncbi.nlm.nih.gov/SNP. Intronic polymorphism rs1467465 is located at position 28083990 of chromosome 1, between *icb-1* exons 4 and 5 and rs12048235 is located at position 28078471 of the same chromosome, in the intron between exons 2 and 3.

Genomic DNA was isolated from 100 μl EDTA-blood after addition of 300 μl of lysis buffer (1% v/v TritonX, 0.32 M Sucrose, 0.01 M Tris (pH 7.5) and 5 mM MgCl_2_) and two-fold centrifugation (13000 g) for 30 seconds. Pellet was resuspended in 50 μl PCR buffer (GoTaq buffer, Promega, Madison, USA) containing 0.5% Tween 20 and 10 mAnson units proteinase K (Merck, Darmstadt, Germany) followed by incubation at 50°C over night and finally heat inactivation of the enzyme for 10 min at 95°C. The genomic DNA-containing lysate was subjected to a tetra-primer ARMS PCR approach [10] allowing allele-specific amplification using the PCR primers listed in Table 2 (synthesized at Metabion, Martinsried, Germany). For this purpose, to 100 ng of genomic DNA, 2 μl of 5 x GoTaq buffer, 0.2 μl of dNTP Mix (10 mM) (Fermentas, St. Leon-Rot, Germany), 0.2 μl of each PCR primer (10 μM) and 0.5 units GoTaq polymerase (Promega, Madison, USA) was added and PCR reaction was carried out using a Biometra T1 thermocycler (Goettingen, Germany). PCR program was 10 min 94°C followed by 38 PCR cycles of 94°C (30 sec), 56°C (30 sec) and 72°C (60 sec), followed by a final extension for 5 min step at 72°C. PCR products were analysed by means of 1.5% agarose gelelectrophoresis.

**Table 2:** PCR primers used for SNP analysis

**Polymorphism**	**Primer**	**Sequence**
rs1467465	IP1	GCAGACGTCATGTAGCATCTGGCCCA
	IP2	GAAAGAGGACTTCCATTGCGTTCCCCAAC
	OP1	AGGGAGCTGTGCCGCACACTCTGTAAAG
	OP2	GGAGACAGGGTGTTCCTGGGATTTTCCA
rs12048235	IP1	GAAGTCTAGTGTGTCTGTCAGGGAATTTG
	IP2	ACCAGAGACACAGAGAATGGAAGAGGTAT
	OP1	GGAGGAAAGGCTAAGATGGCAGTTAG
	OP2	ATGACAGATTTGCATCTGGACATCTG

### Statistical analysis

Deviation from the Hardy-Weinberg equilibrium was estimated by the Fisher’s exact test and the χ^2^ test, and all values were subjected to one-way ANOVA to achieve homogeneity of variance. Statistical tests for association (C.I.: 95% confidence interval) and for significance were carried out using SPSS for Windows 8.0 (SPSS, Inc., Chicago, IL). *P* < 0.01 was considered statistically significant. After tests for deviation from Hardy-Weinberg equilibrium were conducted, allele frequency, allele positiviy and genotype frequencies were determined. Odds ratio (OR) was calculated using the more frequent homozygous genotypes as reference group.

## Results

### Genotype analysis

After genotyping of 184 ovarian cancer patients and such as many women without any malignancy, we observed a higher frequency of heterozygous AG genotype of the *icb-1* gene SNP rs1467465 in the patients group. Genotype-phenotype association suggested that the heterozygous genotype of rs1467465 could be a risk factor for the development of ovarian cancer (OR 1.63, 95% CI 1.045-2.045, p = 0.03) (Table 3). The frequency of the homozygous genotypes did not differ significantly between both groups in this study. Moreover, no significant differences between healthy women and women with ovarian cancer were found for the heterozygous/homozygous frequencies of the *icb-*1 gene SNP rs12048235 in this study (Table 3).

**Table 3:** Comparison of SNP genotypes between ovarian cancer patients and women without any malignancy

**rs1467465**	**Genotype frequency**			**Allele frequency**		**Allele positivity**	
	**GG**	**AA**	**AG**	**G**	**A**	**G**	**A**
**Cases**	0.35	0.15	0.50	0.60	0.40	0.85	0.65
**Controls**	0.47	0.13	0.41	0.67	0.33	0.88	0.53
**P**		0.13	**0.03**		**0.04**		**0.02**
**OR**		n.s.	1.63		1.37		1.632
**C.I. 95 %**		n.s.	1.045-2.045		1.013-1.853		1.072-2.483
**rs12048235**	**Genotype frequency**			**Allele frequency**		**Allele positivity**	
	**GG**	**AA**	**AG**	**G**	**A**	**G**	**A**
**Cases**	0.42	0.12	0.46	0.65	0.35	0.88	0.58
**Controls**	0.42	0.15	0.45	0.63	0.37	0.85	0.59
**P**		0.48	0.87		0.61		0.90
**OR**		n.s.	n.s.		n.s.		n.s.
**C.I. 95 %**		n.s.	n.s.		n.s.		n.s.

After tests for deviation from Hardy-Weinberg equilibrium were conducted, allele frequency, allele positivity and genotype frequencies were determined. Odds ratio (OR) was calculated using the more frequent homozygous genotypes as reference group. Bold p-values were considered to be statistically significant. n.s.=not significant.

### Allele frequency

When we analysed the allele frequencies of the *icb-1* gene SNPs we found that in women with ovarian cancer the A allele of SNP rs1467465 was carried significantly more often than in women without an ovarian malignancy (0.40 vs. 0.33, OR 1.37, 95% CI 1.013-1-853, p = 0.04) (Table 3). Ovarian cancer patients exhibited significantly less G-positive alleles of this SNP. We were not able to show any significant differences between healthy women and women with ovarian cancer in the allele positivity analysis of the *icb-1* gene SNP rs12048235 (Table 3).

### Allele positivity

Phenotype-genotype association analyses of allele positivity of the *icb-1* gene SNPs revealed that A allele positivity of SNP rs1467465 was more frequent in patients with ovarian cancer than in the control group of healthy women (0.65 vs. 0.53, OR 1.632, 95% CI 1.072-2.483, p = 0.02) (Table 3). G positive alleles were less exhibited in patients with ovarian cancer. We did not find any significant difference between healthy women and women with ovarian cancer in allele positivity analyses of the *icb-1* gene SNP rs12048235 (Table 3).

## Discussion

Our data suggest SNP rs467465 in *icb-1* gene to affect susceptibility to ovarian cancer. The vertebrate gene *icb-1* previously has been shown to inhibit growth of ovarian cancer cells in vitro and to suppress expression of ovarian cancer biomarkers like kallikrein-related peptidase 10 and claudin 16 or other cancer-related genes activated by estrogens or TNFα [7]. Furthermore, knockdown of *icb-1* was shown to inhibit induction of differentiation-associated genes like E-cadherin triggered by vitamin D3 or all-trans retinoic acid. Loss of *icb-1* expression previously was sufficient to transform the estrogen-unresponsive ovarian cancer cell line SK-OV-3 into a line exhibiting a strong proliferative response to estrogen stimuli [5]. Icb-1 gene contains an imperfect estrogen response element (ERE) and transcript levels of *icb-1* were shown to be estrogen-responsive in ovarian cancer cells in an estrogen receptor α (ERα)-dependent manner [4]. Estrogens are able to promote ovarian tumor progression [21], which is associated with loss of cellular differentiation. Thus, women carrying the minor frequent allele or genotype of SNP rs1467465, which might lead to decreased icb-1 function, are suggested to be less sensitive to the antitumoral effects of vitamin D3 and all-trans retinoic acid, but more sensitive to the oncogenic effects triggered by estrogens or TNFα.

In this study, we examined intronic polymorphisms without clear functional relevance, because *icb-1* gene does not exhibit SNPs in exons or in transcription factor binding sites. Numerous studies reported an association between intronic SNPs and disease risk [22-24]. Intronic SNPs could affect intronic splicing enhancer or silencer signals and thereby modulate the expression of different splice variants [25]. This report is the first one analysing the association of *icb-1* gene SNPs rs1467465 and rs12048235 with ovarian cancer risk. Recently, we were able to show that the SNP rs1467465 might affect breast cancer susceptibility, which might corroborate the data presented here [26]. The results of the genotype-phenotype association study we performed clearly demonstrated that the heterozygous genotype of SNP rs1467465, the A-allele and A-positive genotypes were more frequent in ovarian cancer patients and thus might be risk factors for this disease. The general low odds ratios reveal that the effects of the gene polymorphism are low, as expected from a complex etiology.

Taken together, the results of this study suggest that a SNP in human *icb-1* gene might be able to affect susceptibility to ovarian cancer. Our data encourage further studies examining the relevance of *icb-1* gene in ovarian cancer and combining analysis of rs467465 with other polymorphisms affecting ovarian cancer risk.

## Competing interest

The authors declare that they have no competing interest.

## Authors’ contributions

SS carried out genotyping, participated in statistical analysis and drafted the manuscript. CL and MC participated in sample preparation and aquisition TF and OO participated in sample aquisition and corrected the manuscript. OT planned the study, participated in correction of the manuscript and statistical analysis. All authors read and approved the final manuscript.
